# Mixed circuit training as a non-pharmacological strategy to improve platelet function and oxidative balance in type 2 diabetes: role of purinergic signaling

**DOI:** 10.1007/s11302-026-10136-8

**Published:** 2026-03-17

**Authors:** Lucas Macedo Chaves, Samantha Nuncio Prestes, André Campos De Lima, Aline Mânica, Daniela Zanini, Sedinei Lopes Copatti, Clodoaldo Antônio De Sá, Andréia Machado Cardoso

**Affiliations:** 1 Medical Course, Universidade Federal da Fronteira Sul, Campus Chapecó, Chapecó City, Brazil; 2 Graduate Program in Biomedical Sciences, Universidade Federal da Fronteira Sul, Curso de Medicina, Campus Chapecó, Chapecó City, SC CEP: 89802-112 Brazil; 3 Graduate Program in Health Sciences, Universidade Comunitária da Região de Chapecó, Chapecó City, Brazil; 4 Programa de Pós-Graduação Em Ciências da Saúde Unochapecó, Chapecó City, Santa Catarina Brazil

**Keywords:** Type 2 diabetes, Purinergic signaling, Oxidative stress, Platelet aggregation, Mixed circuit training, Non-pharmacological therapy

## Abstract

This study investigates the impact of a 16-week mixed circuit training (MCT) program on purinergic signaling and oxidative stress markers in women with type 2 diabetes mellitus (T2DM), focusing on its potential to modulate platelet-related purinergic signaling and oxidative stress markers that are mechanistically linked to platelet activation. A total of 21 women with T2DM and 23 non-diabetic controls, all sedentary and middle-aged, underwent MCT twice weekly. Biochemical, hemodynamic, and oxidative stress parameters, along with platelet ectonucleotidase activity and extracellular ATP levels, were assessed pre- and post-intervention. MCT significantly decreased ectonucleotidase diphosphohydrolase (E-NTPDase) activity for ADP hydrolysis in platelets, along with a reduction in extracellular ATP levels, indicating a modulation of purinergic signaling. Additionally, exercise enhanced antioxidant defenses, increasing glutathione-S-transferase (GST) activity and vitamin C levels, while reducing myeloperoxidase (MPO) activity, a key pro-oxidant enzyme. These changes suggest a shift toward a biochemical profile that may be relevant to thromboinflammatory pathways; however, no direct measures of platelet aggregation were performed. Mixed circuit training emerges as a valuable non-pharmacological strategy for improving oxidative balance and modulating purinergic markers in women with T2DM, warranting future studies including functional platelet assays and clinical outcomes.

## Introduction

Type 2 diabetes mellitus (T2DM) is an epidemic disease with a major impact on healthcare worldwide, leading to lower quality of life and high levels of morbidity and mortality [1]. According to the Atlas of the International Diabetes Federation, in 2021 [2], 10% of adults were living with the disease in the world, which means 537 million people; furthermore, diabetes was the cause of 6.7 million deaths in the same year. Plus, it is estimated that the number of people with T2DM will reach 643 million in 2030 and 783 million in 2045, reinforcing its global impact on deteriorating health and mortality.

A hyperglycemic state resulting from insulin resistance and dysfunction of pancreatic β-cells is characteristic of T2DM [3]. The main causes of morbidity and mortality of this disease are its microvascular complications, such as nephropathy, neuropathy, retinopathy, and erectile dysfunction [4], and also macrovascular complications, like atherosclerosis, vascular inflammation, vasoconstriction, and thrombosis that increase the risk of coronary and cerebrovascular events [5]. Macrovascular complications derive from endothelial cell dysfunction, inadequate fibrinolysis, and elevated platelet activity [6].

In this context, purinergic signaling plays an important role in platelet activation and aggregation. In response to an initial stimulus, platelets secrete granules that contain high concentrations of ATP and ADP, promoting aggregation through P2Y and P2X receptors [7]. With this in mind, a mechanism for controlling platelet activation, thrombus growth, and stability is the enzymes of the purinergic system, capable of controlling circulating levels of ADP and ATP [8].

The enzyme NTPDase is able to hydrolyze ATP and ADP into AMP, while the enzyme ecto-5′-nucleotidase (E-NT5) hydrolyzes AMP, forming adenosine, a potent inhibitor of platelet activation [8]. Among these, ADP stands out as a platelet aggregating agent, and even at micromolar concentrations, this nucleotide demonstrates an important pro-aggregating action in humans [9].

In addition to the components of the purinergic system, reactive oxygen species (ROS) are also of great importance for platelet activation, both for the coagulation cascade and in the fibrinolysis process, making platelets procoagulants and aggregators [10]. These ROS are formed from physiological processes, mainly in mitochondria during the oxidative phosphorylation process, but also by specific enzymes that have their concentration regulated by antioxidant mechanisms [11]. When an imbalance between oxidizing agents and antioxidants happens, it favors the formation of ROS, characterizing oxidative stress, further amplifying platelet hyperreactivity [12].

In the context of T2DM, changes in insulin sensitivity and an increase in plasma glucose concentration expand the production of ROS [13, 14]. Low levels of enzymatic antioxidants have been reported in patients with hyperglycemia and complications related to T2DM [15]. Plus, low levels of non-enzymatic antioxidants and high levels of oxidative damage markers have been found in patients with T2DM [16], highlighting the participation of ROS in the diabetic condition not only by contributing to vascular dysfunction, but also interfering with purinergic enzyme activity.

Indeed, physical exercise can enhance overall health. Strength training alone helps to improve body composition and insulin sensitivity [17], while aerobic exercise demonstrated a reduction in blood glucose and triglyceride levels, as well as systemic blood pressure [18, 19]. Moreover, both aerobic and resistance training act on purinergic signaling through different mechanisms [20]. Therefore, when aerobic and anaerobic exercises are performed alternately in the same session, there is a new type of exercise, called mixed circuit training [21].

It has already been demonstrated that mixed circuit physical training improves hemodynamic and anthropometric parameters, as well as reduces glycemic levels [22], pointing to physical exercise as a non-pharmacological strategy to improve metabolic and inflammatory parameters in T2DM. However, there are no previous studies demonstrating its impact on the activity of purinergic signaling and oxidative stress parameters in women with T2DM.

In this context, our study aims to evaluate the impact of 16 weeks of mixed circuit training on purinergic signaling and oxidative stress components, plus hemodynamic, biochemical, and anthropometric parameters in women with T2DM, thereby identifying potential mechanisms underlying the anti-thrombotic effects of this type of exercise.

## Material and methods

### Sample size calculation

The sample size was determined a priori using G*Power 3.1 software, considering an alpha level of 0.05, statistical power of 0.80, and an effect size derived from Cardoso et al. [9], who evaluated similar purinergic outcomes in exercise-based interventions. This analysis indicated that a minimum of 38 participants (19 per group) would be required to detect significant differences. To ensure adequate power, 67 women were initially recruited (35 in the control group and 32 in the diabetes group). After applying attendance and eligibility criteria during baseline procedures, 27 participants remained in the control group and 24 in the diabetes group. Following the intervention, the final sample consisted of 23 normoglycemic women and 21 women with T2DM, thereby exceeding the minimum sample size required for this study design.

### Participants

The participants of the present study were women, sedentary, aged between 40 and 60 years old. Women diagnosed with T2DM were in the diabetes group (DG) and women without a T2DM diagnosis were in the control group (CG). The female sex was chosen because women are more likely to engage in physical exercise protocols. Men were excluded to avoid heterogeneity in groups.

The participants should meet minimum frequency criteria of at least 75% and not miss four consecutive sessions. Also, alimentary habits should be maintained, and participants should not do concomitant exercises during the training protocol. The final sample consisted of 44 women (21 DG and 23 CG).

The DG (*n* = 21) was composed of sedentary women with the diagnosis of T2DM, which is characterized as fasting glucose above 126 mg/dL or random glucose above 200 mg/dL with unequivocal diabetes symptoms or HbA1c above 6.5% [23] in use of hypoglycemic medications, who were submitted to mixed circuit training. The medications used included metformin (14 subjects), gliclazide plus metformin (5 subjects), insulin aspart–glargine (2 subjects), and gliclazide (1 subject). The CG (*n* = 23) was composed of sedentary women without T2DM diagnosis who performed the same exercise protocol. This study was approved by the Ethics Committee of the Federal University of Fronteira Sul (Protocol number 4.598.914; Clinical trial number: not applicable). Every participant read and signed the consent to participate declaration.

Data collection was carried out at two different times: before the start of the physical training protocol and after 16 weeks of performing the proposed exercise. Platelets and serum were separated and frozen before biochemical analysis, as described below.

### Nutritional questionnaire

The nutritional questionnaire is composed of 18 questions that aim to evaluate and qualify the diet of the person being interviewed, with the objective of this research to evaluate whether the volunteers made changes in their eating habits associated with the proposed physical exercise. The questionnaire was applied in the initial collections and in the collection after the intervention. If dietary changes were noticed during the research period, the participant would be excluded from the statistical analysis.

### Mixed circuit training protocol

The exercise protocol proposed for the participants was a mixed circuit, combining aerobic and resistance exercises in a circuit form (strength exercises followed by an aerobic exercise). The participants attended the gym at Academia Transformação, located in Chapecó-Brazil, and the exercises were conducted by a qualified professional in the field of Physical Education.

The volunteers performed the mixed circuit training twice a week, on non-consecutive days, for 16 weeks, totaling 32 sessions lasting 50 min each. The protocol was divided into four training mesocycles, each consisting of four microcycles (weekly) of training, with undulating periodization; that is, every 4 weeks, the volume and intensity of training were alternated, respecting the following order: Start with high volume and low intensity with a rest of 30 ± 5 s; after 4 weeks, it was changed to high intensity and low volume with a rest of 35 ± 5 s; after, low intensity and high volume with a rest of 30 ± 5 s; and finally, high intensity and low volume with a rest of 35 ± 5 s.

### Anthropometric and hemodynamic parameters assessment

Height, body mass, and waist, hip, and thigh circumference were evaluated according to the recommendations of the International Society for the Advancement of Kinanthropometry (ISAK) [24].

Blood pressure measurements were performed using an Aneroide Premium sphygmomanometer with a resolution of 0–300 mmHg and a maximum circumference of 35 cm. The blood pressure measurements were performed after 15 min of a relaxing state. The blood pressure measurement was performed by a health care professional properly trained.

Body mass index (BMI) was calculated as body mass divided by height squared (kg/m^2^). BMI together with the measurement of waist circumference (WC) allowed us to deduce values indicative of visceral fat, which proved to be an important predictor of glucose homeostasis and mortality [25].

Visceral adipose tissue (VAT) was predicted by the protocol by Samouda et al*.* [26] which uses values of WC, proximal thigh circumference (PTC), age, and BMI and which was validated using computed tomography. In this protocol, the TAV is determined according to the area in cm^2^ by the following equation: TAV = 2.15 × waist circumference − 3.63 × proximal thigh circumference + 1.46 × age + 6.22 × BMI − 92, 713 [SE (R2) = 36.88 (0.836)].

Total muscle mass (MM) was predicted by the protocol by Heymsfield et al*.* [27], validated based on dual emission x-ray densitometry (Dexa). In this case, the MM was determined by the following equation: MM = 0.25 × weight + 0.09 × height − 0.111 × age + 0.0005 × age^2^ − 0.06 × WC + 2 × race − 4.5 [SE (R2) = 1.7 (0.89)]. While the percentage of muscle mass (%MM) was determined by the ratio MM/body weight.

The percentage of body fat mass (%FM) was predicted by the protocol of Lee et al. [28], validated based on Dexa. In this protocol, %FM is determined by the following equation: %FM = 50.46 + 0.07 × age − 0.26 × height + 0.27 × WC ± race [SE (R2) = 3.86 (0.65)]. Total fat mass (FM) was determined by the product of %FM and body weight.

### Isolation of platelets

Platelets were isolated following the method described by Pilla et al. (1996) and modified by Lunkes et al. [29]. Blood was collected in vacuum tubes with 0.126 mol/L sodium citrate and was first centrifuged at 1200 rpm for 10 min to remove blood cells. Afterward, the platelet-rich plasma was centrifuged at 5000 rpm for 30 min and washed twice with 3.5 mmol/L isomolar HEPES buffer for 10 min at 5000 rpm. Finally, platelets were suspended in 500 µL in isomolar HEPES buffer 3.5 mmol/L. The amount of protein was determined by the Bradford method and adjusted using Comassie Blue using bovine albumin as standard to 0.4–0.6 mg/mL.

### Serum isolation

Blood without anticoagulant was centrifuged for 15 min at 3500 rpm to separate the supernatant.

### Determination of ATP levels

To quantitatively determine ATP in serum, the commercial ATP determination Kit (InvitrogenⓇ) was used. ATP is quantified using bioluminescence from recombinant luciferase and its substrate d-luciferin. The assay is based on the need for ATP by luciferase to produce light, which was evaluated at a wavelength of 560 nm.

### NTPDase and ecto-5′-nucleotidase activities determination

The activities of E-NTPDase 1 and E-NT5 were determined by a colorimetric assay that measures the release of inorganic phosphate. The reaction for E-NTPDase was carried out in a medium proposed by Pilla et al. [30] composed of CaCl2 5 mmol/L, NaCl 100 mmol/L, KCl 5 mmol/L, glucose 6 mmol/L, and Tris–HCl buffer 50 mmol/L, pH 7.4. For E-NT5, the system is the same except that the 5 mmol/L CaCl2 is replaced by 10 mmol/L MgCl2. Twenty microliters of platelets (8–12 µg of proteins) suspended in 3.5 mmol/L HEPES were added to the medium and pre-incubated for 10 min at 37 °C. Then, the reaction was started by adding ATP or ADP at 1.0 mmol/L to measure E-NTPDase, and adenosine monophosphate (AMP) at 2.0 mmol/L for E-NT5, and incubated at 37 °C for 60 min. Both reactions were stopped by adding 200 µL of 10% trichloroacetic acid (ATA), providing a final concentration of 5%. The release of inorganic phosphate (Pi) was measured using the method of Chan et al. [31] using malachite green as the dye and KH2PO4 as the standard, with a spectrophotometer reading at 630 nm. Control and patients were analyzed in triplicates. The specific activity of the enzyme was expressed as nmol Pi released/min/mg of protein.

### In vitro analysis on the effect of antidiabetic medications

Patients were undergoing previous treatment for diabetes, and in order to mitigate potential effects of antidiabetic drugs on the outcomes, the enzymatic activity of E-NTPDase was tested in vitro in the presence of antidiabetic medications (metformin and gliclazide). The aim was to assess whether these drugs could influence enzyme activity and thereby represent a potential source of bias in the study results. A total of 15 mL of whole blood was collected in citrate tubes, and platelets were isolated. Different concentrations of the medications, corresponding to those used by the patients (metformin: control, 400 ng/L, 800 ng/L, and 1600 ng/L; gliclazide: 1 ng/L, 1.5 ng/L, and 3 ng/L), were added to the platelets, and purinergic system enzyme assays were performed as described by Pilla et al. [30] for NTPDase and ecto-5′-nucleotidase activities.

### Oxidative stress analysis

Myeloperoxidase (MPO) activity was determined in serum, using the method of Kayyali and colleagues [32]. Enzymatic activity was evaluated by a spectrophotometer using a peroxidase coupling system to a system containing phenol, 4-aminoantipyrine, and H2O2. The results express in µmol the amount of quinoneimine produced in 30 min measured at a wavelength of 492 nm.

Another enzyme evaluated was glutathione-S-transferase (GST). Its antioxidant activity was determined in serum samples by the method of Warholm and collaborators [33], counting on an adequate amount of proteins in the sample, which was determined by the Bradford method and expressed as the delta absorbance of the sample.

Ascorbic acid (or vitamin C) levels were measured in serum, according to the method of Roe and Kuether [34]. In this method, dehydroascorbic acid is coupled to 2,4-dinitrophenylhydrazine, and the resulting derivative is treated with sulfuric acid (H2SO4) to produce a new color that will be measured by a spectrophotometer evaluation at a wavelength of 520 nm.

Total thiols were measured in serum according to the method of Ellman [35], which can be described as the reduction of 5–5′-dithiobis acid (2-nitrobenzoic acid) measured at a wavelength of 412 nm. The results were expressed as µmol T-SH/mL serum. Non-protein thiols were also tested in serum using the method of Ellman [35] with some modifications. Ten percent ATA was added to the serum, and the sample consisted of the supernatant. The reaction was read at 412 nm after adding 5–5′-dithiobis acid (2-nitrobenzoic acid). Results were expressed as µmol NPSH/mL serum.

### Assessment of lipid profile and glycated hemoglobin (HbA1c)

Total cholesterol (TC), high-density lipoprotein (HDL), and total triglycerides (TG) tests were carried out in an outsourced laboratory (Laboratório Diagnósticos do Brasil). Serum was the sample used, and the analysis method was colorimetric. Low-density lipoprotein (LDL) was calculated using the Friedewald formula [36]: LDL = TC − HDL − (TG/5).

HbA1c was also assessed in an outsourced laboratory (Laboratório Diagnósticos do Brasil), using the turbidimetry method. The material used for analysis was blood collected in a tube with EDTA.

### Statistical analysis

First, the data were submitted to the Shapiro-Wilk test to verify its normality. As the data follow a normal distribution, the difference between the means was statistically analyzed by the two-way analysis of variance (ANOVA) using the statistical program Graph Pad Prism version 8.0

## Results

Table 1 shows the hemodynamic characteristics of the groups before and after the intervention. Regarding SBP, there was a reduction in both groups after exercise. It was also possible to observe that the DG had higher post-intervention SBP levels compared to the CG (120.24 ± 7.62 mmHg vs 114.04 ± 9.23 mmHg). As for DBP levels, in the post-intervention period, the DG had higher values than the CG (80.9 ± 5.5 mmHg vs 74.6 ± 6.5 mmHg). DBP levels in the same group showed no difference before and after the intervention.

**Table 1:** Hemodynamic parameters in the control group (CG) and diabetic group (DG) before (pre) and after (post) the application of the circuit mixed training protocol

	Time	CG	DG
SBP (mm/Hg)	Pre	124.9 ± 11.40	129.90 ± 11.94
	Post	114.04 ± 9.23a	120.24 ± 7.62a
	%	−8.70%	−7.40%
DBP (mm/Hg)	Pre	76.6 ± 7.5	81.6 ± 7.6
	Post	74.6 ± 6.5	80.9 ± 5.5b
	%	−2.60%	−1%

Values of systolic blood pressure (SBP) and diastolic blood pressure (DBP) assessed in the control group (CG) and diabetic group (DG), before the application of the circuit mixed training (pre) and after 16 weeks/32 training sessions (post). Data are presented as mean and standard deviation, along with the percentage difference. Statistical analysis was performed using two-way ANOVA, considering *p* < 0.05 (a) statistically significant difference between pre- and post-training within the same group and (b) statistically significant difference between groups at the same time point (*p* < 0.05)

Table 2 presents the anthropometric characteristics by groups before and after the intervention. The variables of weight, BMI, WC, CCO, WHR (waist to height ratio), TAV, MM (kg), and MA (kg) showed no difference pre-intervention and post-intervention in DG and CG, or in the same period between groups. However, in the pre-intervention period, a difference was observed between the groups regarding the variables %MM and %MA, with a higher percentage of MM in the CG compared to the DG (25.10 ± 1.45% vs 23.93% ± 0.86) and a higher percentage of MA in the DG compared to the CG (40.95 ± 2.88% vs 38.03% ± 3.76).

**Table 2:** Anthropometric parameters in the control group (CG) and diabetic group (DG) before (pre) and after (post) the application of the circuit mixed training protocol

	Time	CG	DG
Weight (kg)	Pre	74.95 ± 9.54	77.07 ± 14.53
	Post	74.00 ± 10.07	75.34 ± 14.8
	%	−1.40%	−2.20%
BMI (cm2)	Pre	29.93 ± 4.01	31.31 ± 5.57
	Post	28.76 ± 4.18	31.05 ± 5.46
	%	−3.90%	−0.90%
WC (cm)	Pre	96.74 ± 11.39	102.76 ± 10.30
	Post	96.22 ± 12.25	101.14 ± 9.51
	%	−0.50%	−1.60%
TC (cm)	Pre	61.52 ± 6.08	65.52 ± 5.46
	Post	63.24 ± 6.46	66.48 ± 5.39
	%	2.80%	1.5%
WHR	Pre	0.60 ± 0.08	0.66 ± 0.06
	Post	0.61 ± 0.08	0.64 ± 0.06
	%	1.60%	−3%
VAT (cm2)	Pre	137.57 ± 47.95	173.17 ± 42.66
	Post	133.13 ± 51.66	161.98 ± 37.92
	%	−2.90%	−6.50%
MM (kg)	Pre	18.82 ± 2.64	18.61 ± 3.80
	Post	18.36 ± 2.61	18.64 ± 3.61
	%	−1.60%	+0.16%
MM%	Pre	25.10 ± 1.45	23.93 ± 0.86b
	Post	24.8 ± 1.40	24.38 ± 1.20
	%	−1.19%	+1.9%
AM (kg)	Pre	27.84 ± 4.43	31.83 ± 7.93
	Post	27.54 ± 4.62	31.22 ± 7.53
	%	−1.07%	−1.90%
AM%	Pre	38.03 ± 3.76	40.95 ± 2.88b
	Post	38.14 ± 3.98	40.52 ± 2.61
	%	0.28%	−1.10%

Values of weight in kilograms, body mass index (BMI), waist circumference (WC), thigh circumference (TC), waist-to-height ratio (WHR), visceral adipose tissue (VAT), muscle mass in kilograms (MM), muscle mass percentage (MM%), total adipose mass in kilograms (AM), and adipose mass percentage (AM%) assessed in the control group (CG) and diabetic group (DG), before the application of circuit mixed training (pre) and after 16 weeks/32 training sessions (post). Data are presented as mean and standard deviation, along with percentage differences. Statistical analysis was performed using two-way ANOVA, considering *p* < 0.05 (a) statistically significant difference between pre- and post-training within the same group and (b) statistically significant difference between groups at the same time point (*p* < 0.05)

The results of the biochemical analyses are shown in Table 3. It was possible to notice that in the pre-intervention period, HbA1c levels were increased in the DG and within the appropriate reference values for the control group, confirming that they were not diabetic (6.36 ± 0.67% vs 5.4 ± 0.35%). Regarding lipid profile analyses, no changes were observed when TC, LDL, and HDL were evaluated in both groups after the intervention. However, in the pre-intervention period, a difference was noted in HDL levels, which were higher in the CG compared to the DG (58.22 ± 10.08 mg/dL vs 48.38 ± 10.56 mg/dL). Furthermore, TG levels showed a difference in the post-intervention period, being increased in the DG compared to the CG (139.9 ± 55.6 mg/dL vs 92.77 ± 34.29 mg/dL).

**Table 3:** Biochemical parameters in the control group (CG) and diabetic group (DG) before (pre) and after (post) the application of the circuit mixed training protocol

	Time	CG	DG
HbA1c (%)	Pre	5.4 ± 0.35	6.36 ± 0.67b
	Post	5.5 ± 0.31	6.43 ± 0.58
	%	1.80%	1.1%
TC (mg/dL)	Pre	208.7 ± 47.32	189.57 ± 37.82
	Post	201.4 ± 44.09	190.48 ± 34.26
	%	−3.50%	0.60%
HDL (mg/dL)	Pre	58.22 ± 10.08	48.38 ± 10.56b
	Post	57.52 ± 10	50.57 ± 9.3
	%	−1.20%	+4.50%
LDL (mg/dL)	Pre	125.43 ± 37.58	107.84 ± 32.38
	Post	124.03 ± 35.22	106.43 ± 30.23
	%	−1.10%	−1.30%
TG (mg/dL)	Pre	120.3 ± 44.64	146.3 ± 54.5
	Post	92.77 ± 34.29	139.9 ± 55.6b
	%	−22.90%	−4.4%

Values of glycated hemoglobin (HbA1c), total cholesterol (TC), high-density lipoprotein (HDL), low-density lipoprotein (LDL), and triglycerides (TG) assessed in the control group (CG) and diabetic group (DG), before the application of circuit mixed training (pre) and after 16 weeks/32 training sessions (post). Data are presented as mean and standard deviation, along with percentage differences. Statistical analysis was performed using two-way ANOVA, considering *p* < 0.05 (a) statistically significant difference between pre- and post-training within the same group and (b) statistically significant difference between groups at the same time point (*p* < 0.05)

**Fig. 1:**
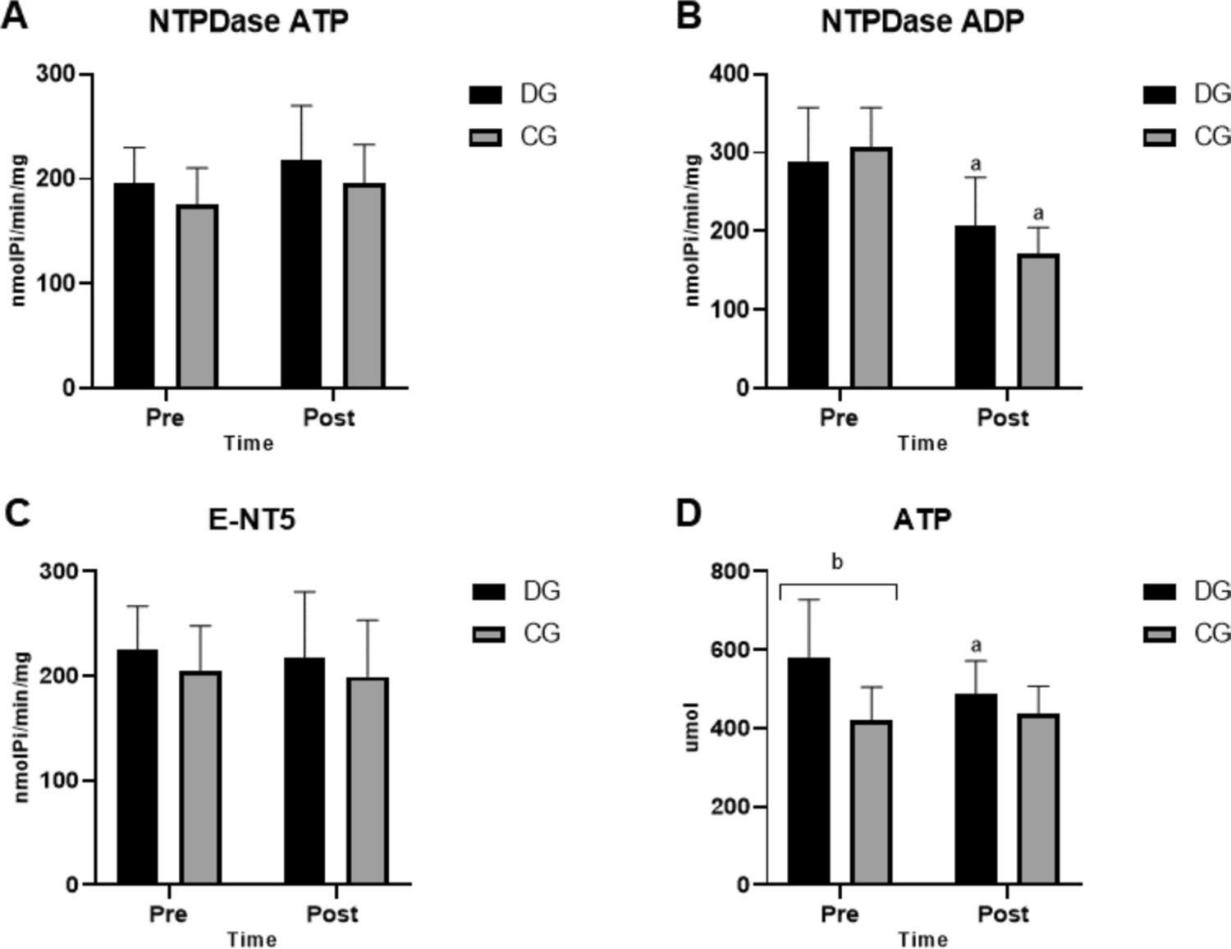
Ectonucleotidase activity and extracellular ATP levels before (pre) and after (post) the application of circuit mixed training. CG, control group; DG, diabetes group. **A** E-NTPDase activity for ATP hydrolysis. **B** E-NTPDase activity for ADP hydrolysis. **C** E-NT5 activity for AMP hydrolysis. **D** Serum ATP concentration. Data are presented as mean and standard deviation. Statistical analysis was performed using two-way ANOVA, considering *p* < 0.05. (a) statistically significant difference between pre- and post-training within the same group and (b) statistically significant difference between groups at the same time point (*p* < 0.05)

The evaluation of ectonucleotidases is shown in Fig. 1. Regarding the activity of E-NTPDase, it can be seen that in the DG, ADP hydrolysis (Fig. 1B) was reduced comparing pre-training and post-training (289.2 ± 68.18 nmolPi/min/mg protein vs 207.1 ± 61.38 nmolPi/min/mg protein). For this same parameter, a decrease in enzyme activity was also noted in CG pre- and post-intervention (307.6 ± 49.53 nmolPi/min/mg protein vs 172 ±32.73 nmolPi/min/mg protein). However, no changes were observed in E-NTPDase activity when the substrate was ATP (Fig. 1A) or in E-NT5 activity (Fig. 1C).

Regarding the concentration of extracellular ATP (Fig. 1D), before the physical training protocol, levels in the DG were significantly higher than in the CG (581.4 ± 146.2 vs 421.5 ± 83.29; *p* < 0.0001). Furthermore, after 16 weeks of mixed circuit exercise, there was a reduction in the concentration of extracellular ATP in the DG compared to pre-intervention (581.4 ± 146.2 vs 487.4 ± 84.16; *p* < 0.05). No changes in CG were observed after physical exercise.

Patients were using a variety of antidiabetic medications prior to enrollment in the study, and in order to avoid major metabolic imbalances, the decision was to maintain their preexisting medication regimens. The medications used included metformin (66.66% of patients), gliclazide plus metformin (23.81% of patients), insulin aspart–glargine (9.52% of patients), and gliclazide (4.76% of patients). The in vitro assays performed with metformin and gliclazide at different concentrations (metformin: control, 400 ng/L, 800 ng/L, and 1600 ng/L; gliclazide: control, 1 ng/L, 1.5 ng/L, and 3 ng/L) did not demonstrate any differences in enzymatic activities (data not shown).

**Fig. 2:**
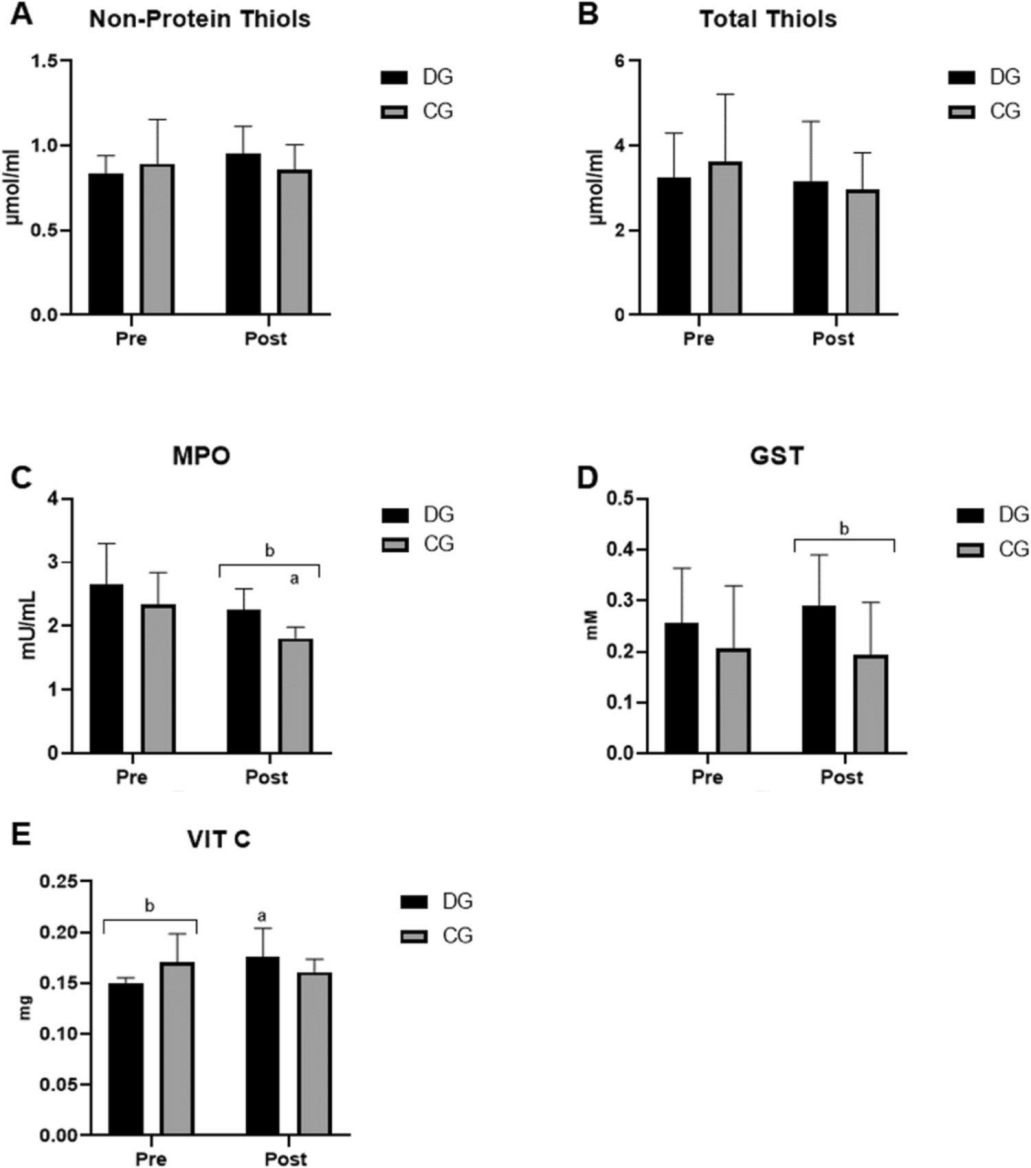
Oxidative stress parameters before (pre) and after (post) the application of circuit-based mixed training. CG, control group; DG, diabetes group. **A** Total thiol concentration in serum. **B** Non-protein thiol concentration in serum. **C** Myeloperoxidase (MPO) activity. **D** Glutathione-S-transferase (GST) activity. **E** Vitamin C levels. Data are presented as mean and standard deviation. Statistical analysis was performed using two-way ANOVA, with *p* < 0.05 indicating (a) statistically significant differences between pre- and post-training within the same group and (b) statistically significant differences between groups at the same time point (*p* < 0.05)

About the oxidative stress parameters evaluated, there were favorable changes post-intervention with mixed circuit physical training (Fig. 2). An increase in the antioxidant defenses GST and vitamin C was noted, at the same time as a decrease in MPO activity, both with statistical significance.

Initially, the levels of protein thiols and non-protein thiols were quantified. Figure 2A demonstrates the levels of protein thiols in the DG and CG before and after physical exercise, which did not show a difference between groups or before and after the intervention. Figure 2B demonstrates the levels of non-protein thiols in the DG and CG before and after physical exercise; there were also no differences between groups or before and after the intervention.

Figure 2C shows MPO activity in the DG and CG before and after physical exercise. It can be observed that in the DG, MPO activity decreased after the intervention (2.65 ± 0.645 mU/mL vs 2.254 ± 0.33 mU/mL). Also in the CG, a decrease in MPO activity was observed post-training (2.343 ± 0.4677 mU/mL vs 1.812 ± 0.1738 mU/mL). Furthermore, when comparing the two groups post-intervention, it was possible to observe decreased MPO activity in the CG compared to the DG.

Another antioxidant evaluated was GST, and its activity is represented in Fig. 2D. When comparing the groups after the intervention, an increase in enzyme activity was noted in the DG compared to the CG (0.2916 ± 0.098 mM vs 0.1941 ± 0.1022 mM). Regarding vitamin C levels, at the pre-intervention period, the CG had higher vitamin C values compared to the DG (0.1706 ± 0.0278 mg vs 0.1492 ± 0.0061 mg). It was also possible to notice an increase in vitamin C levels when comparing the pre-intervention and post-intervention DG (0.1492 ± 0.0061 mg vs 0.1760 ± 0.0281 mg). There were no changes in CG after the physical training protocol.

## Discussion

Considering the importance of the purinergic system and oxidative stress in modulating platelet activity and the fact that physical exercise is capable of modifying its parameters in a useful way, this study investigated the effects of mixed circuit physical training on the activity of ectonucleotidases in platelets, serum ATP levels, and serum oxidative stress components. Hemodynamic, anthropometric, and biochemical parameters (concerning the glycemic and lipid profile) were also considered.

Although the present study provides important insights into how type 2 diabetes modulates the platelet and redox responses to mixed circuit training, we recognize a methodological limitation regarding the absence of a non-exercising control group. Both the diabetic and normoglycemic groups underwent the same training protocol; therefore, our findings do not allow conclusions about the isolated efficacy of exercise compared to a sedentary condition. Instead, the study was designed to evaluate differential physiological adaptations to exercise between distinct glycemic profiles. All participants were sedentary at baseline, and pre-intervention measurements served as internal controls, minimizing interpersonal variability and preventing the introduction of additional confounding factors that would arise from separate non-exercising groups. This approach is frequently adopted in exercise physiology research and is appropriate for investigating relative changes induced by training within the same individuals. Nevertheless, we acknowledge that the absence of a non-exercising group limits causal inference regarding exercise per se, and this should be taken into account when interpreting the results. Future studies should incorporate an additional sedentary control arm to determine the absolute contribution of exercise to the observed effects.

Regarding hemodynamic parameters, it is already established in the literature that T2DM decompensation modifies blood pressure levels [37], increasing the risk of cardiovascular events. The results of this study are in agreement with the already known [38–41] impacts of physical exercise on hemodynamic aspects found in the literature. SBP analysis showed a significant decrease after the mixed circuit exercise protocol, especially in the diabetes group. In contrast, DBP did not change significantly after 16 weeks of training.

Meanwhile, it is expected that individuals without T2DM will present an HbA1c < 6, which is in line with what was observed in our control group both pre- and post-intervention. HbA1c reflects the body’s glycemic levels in the last 3 months, being an important tool for assessing blood glucose levels in T2DM [42]. In the pre-intervention period of our study, HbA1c had increased values in the DG compared to the CG, which was already expected and reinforced that participants from the control group did not have T2DM.

About the lipid profile, it is known that elevated LDL and TG levels increase the risk of cardiovascular disease [43], at the same time that T2DM also expands the risk of these events [44]. This reinforces the importance of following the therapeutic goals of TC, HDL, LDL, and TG levels recommended by the American Diabetes Association (ADA) [45].

In our study, a tendency to increase TC levels in the DG was observed, which may be associated with an expansion in HDL that is useful for removing excess LDL deposited in the blood vessels. This improvement can be strongly associated with physical exercise, considering that the volunteers did not make dietary interventions, which were evaluated through questionnaires about eating habits applied before and after physical training. Concerning TG values, there was no change with statistical significance after the physical training protocol. In this case, dietary modifications associated with physical exercise are strongly recommended to reduce TG rates [43].

Related to the anthropometric results, no significant reductions were observed in body weight, BMI, WC, CCO, TAV, MM, and MA after 16 weeks of mixed circuit physical training. Although a percentage tendency in decreasing TAV can be observed in both groups, this is an important result considering the contribution of adipose tissue to systemic insulin resistance, especially through the production of free fatty acids [46]. Studies that carried out intervention for a longer period [47] or with a greater frequency of physical exercise [48, 49] showed significant improvement in the anthropometric variables analyzed.

Regarding the parameters of the purinergic system evaluated in platelets, it has been proved that nucleotides and nucleosides play an important role in thrombotic regulation. ADP is one of the main promoters of platelet aggregation, activating platelets and causing them to adhere to the initial layer, expanding the thrombus [50]; this role is already well established and important antiplatelet agents act in this process, inhibiting P2Y receptors on platelets [51]. Adenosine is an inhibitor of this process through A2 receptors, increasing the intracellular concentration of cyclic AMP (cAMP) and, as a result, inhibiting platelet activation [52]. Within this context, the enzymes NTPDase and NT5 perform an important role in regulating platelet activity by modulating the presence of ADP and adenosine in the extracellular environment.

The literature shows that people with T2DM have increased E-NTPDase and E-NT5 activity, probably as a compensatory mechanism for the increase in nucleotides in the extracellular environment [53]. Physical exercise directly affects purinergic signaling and consequently, the activity of enzymes that hydrolyze nucleotides. Moreover, it has been observed that, acutely, exercise increases the capacity of these enzymes, which hydrolyze ATP, ADP, and AMP into adenosine [20, 54].

Findings from Martins et al. [55] demonstrated that the usual modifications from metabolic syndrome that precedes T2DM were reversed with regular physical activity, showing a decrease in the activity of E-NTPDase and E-NT5; also, a decline in coagulability modifications related to exercise. It is also important to highlight that the reduction in E-NTPDase activity might involve distinct regulatory mechanisms beyond direct downregulation. It is possible that MCT reduced platelet activation and turnover, leading to lower expression of the enzyme on the platelet surface. Although molecular expression of purinergic enzymes was not evaluated here, future studies should include protein expression analyses to clarify the regulatory pathways involved.

Furthermore, the thrombus microenvironment is composed of other cells such as the ones from the immune system and, mainly, endothelial cells, which also play a regulatory role in platelet activity. In this sense, it was demonstrated the importance of ectonucleotidases in endothelial cells to inhibit platelet activation and aggregation [56], making it necessary, in the future, to study how mixed circuit training affects these cells.

Still on the purinergic signaling, about serum ATP levels, the concentration of this nucleotide significantly increased in the diabetes group compared to the control before the intervention was performed, reinforcing that diabetes promotes an increase in ATP, activating more P2X type receptors [57]. These receptors are capable of regulating platelet activation, both directly, via P2X1 (promoting platelet activation) and P2X7 (inhibiting platelet activation), and indirectly by activating immune system cells that promote platelet activation [58].

In this study, no change in the activity of ectonucleotidases was observed between groups before the intervention protocol. However, after the mixed circuit training exercise, a decrease in E-NTPDase activity for the hydrolysis of ADP was observed, as supported in the literature. Yet, the activity of E-NTPDase for the hydrolysis of ATP and E-NT5 did not change.

It is important to highlight that both ATP and ADP exert pro-aggregatory effects on platelets, although through distinct purinergic receptors. ATP activates P2X1 receptors, inducing rapid calcium influx and platelet shape change, while ADP acts primarily via P2Y receptors to sustain aggregation. Therefore, reductions in extracellular ATP levels—such as those observed after MCT in the diabetic group—may attenuate upstream substrate availability for ADP generation and diminish ATP-mediated platelet activation pathways. This mechanistic interplay helps explain how a decrease in E-NTPDase activity for ADP hydrolysis can coexist with a reduction in ATP concentration without indicating a pro-thrombotic shift.

Moreover, enzymatic activity within the purinergic pathway typically responds to fluctuations in substrate concentration, a phenomenon consistently demonstrated in experimental studies. Thus, the observed reduction in E-NTPDase activity may reflect a physiological adaptation to lower extracellular ATP availability, rather than a mechanism favoring platelet aggregation. Taken together, these findings suggest that MCT induces a coordinated modulation of purinergic signaling elements, favoring reduced nucleotide-driven platelet activation despite decreased ADP hydrolysis.

On the other hand, after mixed circuit training, there was a significant decrease in ATP concentration in the diabetes group, demonstrating that exercise is an important regulator of the purinergic system, considering that a decrease in ATP concentration results in lower activity of P2X receptors that exacerbate platelet activity [58]. Furthermore, the decrease in the extracellular concentration of ATP associated with a decrease in the activity of enzymes demonstrates a balance of the components of the purinergic system, characterized by a change in the pattern of this signaling as a whole.

Although E-NTPDase activity decreased after MCT, extracellular ATP levels were also reduced, which may appear contradictory at first. However, extracellular ATP is determined not only by its degradation but primarily by its release [53]. MCT likely reduced ATP release by lowering basal platelet activation and decreasing inflammatory and oxidative stimuli that trigger ATP efflux. Therefore, even with a reduction in E-NTPDase activity, the net extracellular ATP concentration decreased due to lower cellular release. This suggests that MCT decreases pro-thrombotic signaling independently of enzymatic degradation.

In addition, the association of the purinergic system with oxidative stress seems to play an important role in the prothrombotic state of T2DM. Lipid peroxidation of the phospholipid membrane [59], characteristic of states with high oxidative stress, causes dysfunction of the NTPDase enzyme, reducing its activity [60].

Regarding the oxidative stress parameters evaluated, thiols are non-enzymatic antioxidants that help in the structural protection of cells. In the literature, it was observed that in T2DM, there is a reduction of thiols, and this is correlated with an increase in glycation products, which are markers of advancement and the presence of complications [61]. However, in our results, it was not possible to observe a statistically significant change in this parameter between groups or before and after the intervention.

Another antioxidant evaluated was GST, an enzyme that acts by suppressing the formation of free radicals that accentuate oxidative stress [62]. Studies already demonstrate the increase of GST-dependent antioxidant defenses after resistance and sprint training protocols, so their levels can be associated with physical activity [63, 64]. Furthermore, it has also been demonstrated that GST can play a role in inhibiting platelet aggregation induced by ADP [65]. Similarly, in our study, both groups increased GST activity after the mixed circuit training protocol.

Vitamin C (or ascorbic acid) is an important antioxidant that eliminates ROS [16]. In the literature, it has been demonstrated that, in addition to its antioxidant effect, vitamin C intensifies the formation of prostaglandin E1. This metabolite increases the action of insulin and also plays an antiplatelet action [66], which reinforces the importance of increasing levels of this compound in people with T2DM.

However, when searching for information about the impact of physical exercise on vitamin C, most studies added oral vitamin C supplementation to the training protocols [67–69], and studies that demonstrate the effect of physical exercise alone are hard to find, reiterating the importance of the results found in our study. In this context, after 16 weeks of circuit training, the diabetes group showed a significant increase in vitamin C levels, without its exogenous supplementation.

The activity of MPO was evaluated, an oxidizing enzyme that produces reactive species that attack and modify the function of healthy cells [70]. Its activity showed a significant decrease after 16 weeks of mixed circuit training, especially in the diabetes group. Also, it has already been reported in the literature that increased MPO activity is associated with higher glucose and HbA1c levels [71] and that it can interact with and activate platelets [72]. So, like ADP, it acts in favor of platelet aggregation, predisposing pro-thrombotic events, which explains its importance in the context of T2DM.

This study was not randomized, as the allocation to groups was defined by the presence or absence of type 2 diabetes, a pre-existing clinical condition. Although this design allowed for the evaluation of differential physiological responses to the same exercise protocol, it may introduce selection bias and limit causal inference. Therefore, the findings should be interpreted considering this methodological constraint.

Finally, our findings reveal consistent modulation of purinergic and oxidative pathways, although the absence of multivariate approaches limits a more integrated interpretation of these variables. Future studies should incorporate multidimensional analysis to explore whether these changes correlate directly and to provide a deeper understanding of the mechanisms underlying the effects of MCT.

## Conclusion

In conclusion, mixed circuit training modulated key elements of purinergic signaling and oxidative stress in women with T2DM, including reductions in extracellular ATP levels and MPO activity, and increases in GST activity and vitamin C. These findings suggest a shift toward a biochemical profile associated with lower thrombotic potential; however, no direct measures of platelet aggregation were performed. Therefore, while the results indicate mechanisms that may contribute to improved platelet regulation, functional assays are required to confirm whether these biochemical changes translate into reduced platelet aggregation. Mixed circuit training emerges as a promising non-pharmacological strategy worthy of further investigation in this context.

## Data Availability

Data is provided within the manuscript or upon request.
